# Surgical resection margin for T3–T4 primary acral melanoma: a multicenter retrospective cohort study

**DOI:** 10.1007/s00403-023-02609-2

**Published:** 2023-03-29

**Authors:** Wei Sun, Yu Xu, XingLong Qu, YongJia Jin, ChunMeng Wang, WangJun Yan, Yong Chen

**Affiliations:** 1 Department of Musculoskeletal Surgery, Fudan University Shanghai Cancer Center, Shanghai, 200032 People’s Republic of China; 2 Department of Oncology, Shanghai Medical College, Fudan University, Shanghai, 200032 People’s Republic of China; 3 Department of Oncological Surgery, Minhang Branch, Shanghai Cancer Center, Fudan University, Shanghai, 200240 People’s Republic of China; 4 Shanghai Electric Power Hospital, Shanghai, 200050 People’s Republic of China

**Keywords:** Acral melanoma, Resection margin, Survival, Cutaneous melanoma, Breslow index

## Abstract

Although the National Comprehensive Cancer Network (NCCN) guidelines include clear recommendations for the appropriate resection margins in non-acral cutaneous melanoma, the required margin for acral melanoma is controversial. In this retrospective study, we aimed to investigate whether narrow-margin excision is warranted for thick acral melanoma. Records from 277 melanoma patients with stage T3–T4 disease who underwent radical surgery in three centers in China from September 2010 to October 2018 were reviewed. Clinicopathologic data, including age, gender, excision margin (1–2 cm versus ≥ 2 cm), Clark level, Breslow thickness, ulceration, N stage and adjuvant therapy, were included for survival analysis. The patients were followed up until death or March 31, 2021. Log-rank and Cox regression analyses were used to identify prognostic factors for overall survival (OS), disease-free survival (DFS) and local and in-transit recurrence-free survival (LITRFS). Among all enrolled patients, 207 (74.7%) had acral melanoma, and 70 (25.3%) had non-acral cutaneous melanoma. No significant difference in baseline characteristics was identified between non-acral and acral melanoma, except for age (*p* = 0.004), gender (*p* = 0.009) and ulceration (*p* = 0.048). In non-acral melanoma, a resection margin of 1–2 cm was a poor independent prognostic factor for OS [*p* = 0.015; hazard ratio (HR) (95% CI), 0.26 (0.009–0.77)] and LITRFS [*p* = 0.013; HR (95% CI), 0.19 (0.05–0.71)] but not for DFS [*p* = 0.143; HR (95% CI), 0.51 (0.21–1.25)]. Forty-three (20.8%) patients in the acral melanoma group had a 1–2-cm resection margin. The resection margin was not correlated with patients’ OS (*p* = 0.196 by log-rank analysis, *p* = 0.865 by multivariate survival analysis), DFS (*p* = 0.080 by log-rank analysis, *p* = 0.758 by multivariate survival analysis) or LITRFS (*p* = 0.354 by log-rank analysis) in acral melanoma. As recommended in the NCCN guidelines, a resection margin ≥ 2 cm is required for non-acral cutaneous melanoma. Meanwhile, a narrow resection margin (1–2 cm) may be safe for patients with acral melanoma.

## Introduction

Malignant melanoma is an increasingly common cancer worldwide and primarily arises on the skin [1]. Complete excision of primary melanoma with an appropriate surgical margin is critical for tumor control. Numerous randomized controlled studies have been conducted to determine the optimal surgical margin that can maximally reduce surgical trauma of patients without increasing the probability of tumor recurrence [2–5]. A large randomized controlled study enrolled 900 patients and revealed that the 1-cm excision group had a higher risk of locoregional recurrence, while overall survival was similar to that in the 3-cm excision group [6]. Recently, with a long median follow-up of 6.7 years, another multicenter randomized trial showed that a narrow excision margin (2 cm vs. 4 cm) for patients with thick (> 2 mm) localized cutaneous melanomas did not affect melanoma-specific or overall survival [7]. These findings support the latest National Comprehensive Cancer Network (NCCN) guidelines for cutaneous melanoma surgical margins according to different Breslow thicknesses: 5 mm for in situ melanoma, 1 cm for T1 melanoma, 1–2 cm for T2 melanoma, and 2 cm for T3 and T4 melanoma [8]. However, these conclusions are mostly based on non-acral cutaneous melanoma. Evidence to guide the proper excision margin in acral melanoma patients is currently insufficient and controversial.

Acral melanoma is rare in Caucasians but is the most common subtype in Asians and Africans [9] and considerably differs from cutaneous melanoma, predominantly occurring in sun-protected areas such as the palms, soles and nail beds and having a poorer prognosis and unique genetic background [10, 11]. Histopathologically, most acral melanomas are the acral lentiginous subtype. Compared to non-acral cutaneous melanoma with a higher number of UV-induced mutations, acral melanoma has fewer point mutations, including *BRAF V600* mutations, instead harboring increased numbers of structural rearrangements and amplifications [12–14]. Different genetic backgrounds may explain the different biological behaviors of acral melanoma. Hence, distinctive therapeutic strategies, including drugs and surgical treatment, are required for acral melanoma. Acral melanoma patients are more sensitive to the size of the resection margin. Extensive excision for primary melanoma in the nail beds, fingers and toes leads to amputation. For melanoma in weight-bearing areas, such as the soles or palms, reconstruction requires more extensive skin grafting, leading to greater surgical trauma and long-lasting pain [15]. Therefore, determining the optimal minimum resection margin for acral melanoma, especially for those with a high Breslow thickness, considering both survival and quality of life (QoL) is essential.

The current retrospective multicenter study aimed to determine whether narrow-margin excision is warranted for thick acral melanoma and to investigate the clinicopathologic factors associated with better local control and longer survival in thick acral melanoma. A cutaneous melanoma group was set as the control group.

## Patients and methods

### Patients and clinicopathologic data

Consecutively adult patients with T3–T4 stage (Breslow thickness > 2 mm) cutaneous melanoma who underwent radical surgery in three centers in China (FUSCC, Minhang Branch of FUSCC and Shanghai Electric Power Hospital) from September 2010 to October 2018 were reviewed. Sentinel lymph node biopsy (SLNB) was performed in patients with no clinically positive nodes according to NCCN guidelines for cutaneous melanoma [8]. All patients with positive SLNs underwent complete lymph node dissection (CLND) within 1 month. Therapeutic lymph node dissection (TLND) was performed for patients with clinically positive lymph nodes. Clinicopathologic data, including primary site, age, sex, excision margin (1–2 cm VS ≥ 2 cm), Clark level, Breslow thickness, ulceration, N stage and adjuvant therapy, were included for survival analysis. Pathologic nodal (pN) stage and pathological stage were defined according to the 8th edition of the American Joint Committee on Cancer (AJCC) cancer staging manual [16].

### Procedures for the excision margin

The primary tumors have underwent an complete excisional biopsy (margin of less than 3 mm as recommended of the NCCN guidelines) before the radical surgery, or an immediate radical excision if melanoma was strongly suspected. The excision margin was recorded in the operative note as the surgical distance from the edge of the tumor or the center of the scar (minus 3 mm) in cases of complete lesion excision for biopsy to the closest peripheral cutaneous edge, which was measured and photographed before making an incision. The skin incision was continued vertically down to the deep fascia. Patients underwent direct primary closure or reconstructive surgery with a local flap or a skin graft according to the specific condition of the wound.

### Follow-up

Patients were followed up until death or March 31, 2021. The survival of patients was censored at the date of the last follow-up (March 31, 2021). Patients younger than 18 years or with a follow-up of less than 1 month were excluded. OS was calculated as the interval between radical surgery and death/last follow-up. Disease-free survival (DFS) was defined as the time from radical surgery to local recurrence/distant metastasis or death. Either the time to first local or in-transit recurrence or the time to melanoma-related death was used to calculate local and in-transit recurrence-free survival (LITRFS), whichever occurred first. Recurrence or metastasis was confirmed by pathology or imaging follow-up.

### Statistical analysis

Pearson’s chi-squared test or Fisher’s exact test was performed for univariable analysis of the different resection margin groups. Kaplan–Meier estimation, log-rank analysis and Cox regression analysis were used to identify prognostic factors for OS, DFS and LITRFS. Variables with *p* < 0.2 in the univariable survival analysis were included in the multivariable Cox regression analysis to identify independent prognostic factors and to calculate hazard ratios (HRs) and 95% confidence intervals (95% CIs). All statistical analyses were performed using Statistical Product and Service Solutions (SPSS, version 25.0; SPSS Company, Chicago, IL) software. *p* values less than 0.05 were considered statistically significant.

## Results

### Baseline characteristics

Among all 277 enrolled patients, 207 (74.7%) had acral melanoma, and 70 (25.3%) had non-acral cutaneous melanoma. A total of 148 patients (53.4%) were male, and 129 (46.6%) were female. Fifty-four (77.1%) patients in the non-acral group and 142 (68.6%) in the acral group received chemotherapy and/or high-dose interferon adjuvant therapy. The mean age was 60.14 years (range 20–93). One hundred fifty-four patients (55.6%) had stage T3 disease, and 123 (44.4%) had stage T4 disease. No significant difference in baseline characteristics was identified between the non-acral and acral melanoma, except for age (*p* = 0.004), gender (*p* = 0.009) and ulceration (*p* = 0.048) (Table 1). Acral melanoma patients tended to be older and male and to have ulcerations.

**Table 1:** Baseline characteristics of the acral and non-acral cutaneous melanoma patients

Variable	Non-acral (n = 70)	Acral (n = 207)	Total	Pearson χ^2^	*p*
Age				10.519	**0.001**
< 60	43 (61.4%)	81 (39.1%)	124 (44.8%)		
≥ 60	27 (38.6%)	126 (60.9%)	153 (55.2%)		
Gender				6.790	**0.009**
Male	28 (40.0%)	120 (58.0%)	148 (53.4%)		
Female	42 (60.0%)	87 (42.0%)	129 (46.6%)		
Breslow index (mm)				0.336	0.562
2.01–4	41 (58.6%)	113 (54.6%)	154 (55.6%)		
> 4	29 (41.4%)	94 (45.4%)	123 (44.4%)		
Clark level				1.494	0.684
II	0 (0.0%)	2 (1.0%)	2 (0.7%)		
III	7 (10.0%)	14 (6.8%)	21 (7.6%)		
IV	43 (61.4%)	127 (61.4%)	170 (61.4%)		
V	20 (28.6%)	64 (30.9%)	84 (30.3%)		
Ulceration				3.899	**0.048**
Absent	41 (58.6%)	93 (44.9%)	134 (48.4%)		
Present	29 (41.4%)	114 (55.1%)	143 (51.6%)		
N stage				0.170	0.982
0	39 (55.7%)	116 (56.0%)	155 (56.0%)		
1	10 (14.3%)	32 (15.5%)	42 (15.2%)		
2	11 (15.7%)	33 (15.9%)	44 (15.9%)		
3	10 (14.3%)	26 (12.6%)	36 (13.0%)		
Adjuvant therapy				1.845	0.174
No	16 (22.9%)	65 (31.4%)	81 (29.2%)		
Yes	54 (77.1%)	142 (68.6%)	196 (70.8%)		

Bold font mean *p* value <0.05

As shown in Table 2, 63 (22.7%) patients had a resection margin of 1–2 cm, and 214 (77.3%) patients were in the ≥ 2-cm margin group. Both margin groups were generally matched in terms of baseline characteristics except for Breslow thickness and adjuvant therapy. Patients with thicker melanoma (> 4 mm) tended to receive ≥ 2-cm margin resection (*p* = 0.021) and adjuvant therapy (*p* = 0.038).

**Table 2:** Comparison of clinical Characteristics of the two groups in different margins

Variable	1–2 cm margin group (n = 63)	≥ 2 cm margin group (n = 214)	Pearson χ^2^	*p*
Age			1.913	0.167
< 60	33 (52.4%)	91 (42.5%)		
≥ 60	30 (47.6%)	123 (57.5%)		
Gender			1.794	0.180
Male	29 (46.0%)	119 (55.6%)		
Female	34 (54.0%)	95 (44.4%)		
Breslow index (mm)			5.293	**0.021**
2.01–4 m	43 (68.3%)	111 (51.9%)		
> 4	20 (31.7%)	103 (48.1%)		
Clark level			2.012	0.570
II	0 (0.0%)	2 (0.9%)		
III	6 (9.5%)	15 (7.0%)		
IV	35 (55.6%)	135 (63.1%)		
V	22 (34.9%)	62 (29.0%)		
Ulceration			0.179	0.672
Absent	29 (46.0%)	105 (49.1%)		
Present	34 (54.0%)	109 (50.9%)		
N stage			1.480	0.687
0	38 (60.3%)	117 (54.7%)		
1	10 (15.9%)	32 (15.0%)		
2	7 (11.1%)	37 (17.3%)		
3	8 (12.7%)	28 (13.1%)		
Adjuvant therapy			4.296	**0.038**
No	25 (39.7%)	56 (26.2%)		
Yes	38 (60.3%)	158 (73.8%)		
Subgroup			1.811	0.178
Cutaneous	20 (31.7%)	50 (23.4%)		
Acral	43 (68.3%)	164 (76.6%)		

Bold font mean *p* value <0.05

### Non-acral cutaneous melanoma

**Fig. 1:**
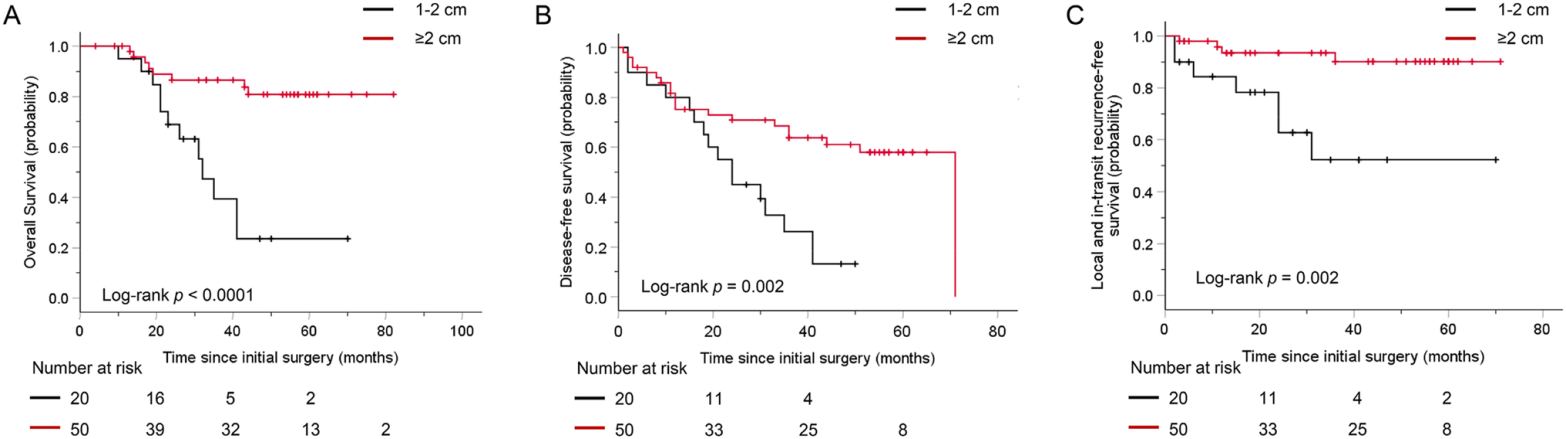
Kaplan–Meier plot curves in patients with cutaneous melanoma with different resection margins. (**A**) Overall survival (*p* < 0.0001). (**B**) Disease-free survival (*p* = 0.002). (**C**) Local and in-transit recurrence-free survival (*p* = 0.002)

In the non-acral cutaneous melanoma group, 28 patients (40.0%) were male, and 27 patients were older than 60 years. Ulceration was found in 29 (41.4%) patients, and 29 patients (41.4%) had a Breslow thickness greater than 4 mm (Table 1). As shown in Fig. 1, twenty (28.6%) patients with a resection margin between 1 and 2 cm had poorer OS (log-rank *p* < 0.0001), DFS (log-rank *p* = 0.002) and LITRFS (log-rank *p* = 0.002). In the multivariate survival analysis, resection margin was an independent prognostic factor for OS [Table 3; *p* = 0.015; hazard ratio (HR) (95% CI), 0.26 (0.009–0.77)] and LITRFS [Table 5; *p* = 0.013; HR (95% CI), 0.19 (0.05–0.71)] but not for DFS [Table 4; *p* = 0.143; HR (95% CI), 0.51 (0.21–1.25)]. Traditional prognostic factors, such as a Breslow thickness greater than 4 mm, a Clark level of V, ulceration and a higher N stage, still reflected the highest risk to OS and DFS. Although adjuvant therapy (chemotherapy and/or high-dose interferon) prolonged DFS in patients with non-acral cutaneous melanoma (*p* = 0.010, Table 4), it was not an independent prognostic factor for OS, DFS or LITRFS.

**Table 3:** Univariate and multivariate OS analysis of the patients in the acral and non-acral cutaneous melanoma

Variable	Overall survival					
	Non-acral cutaneous melanoma			Acral melanoma		
	Univariate analysis	Multivariate analysis		Univariate analysis	Multivariate analysis	
	*p*	HR (95% CI)	*p*	*p*	HR (95% CI)	*p*
Age	0.099	0.89 (0.31–2.55)	0.827	0.284	Not included	
Gender	0.841	Not included		0.511	Not included	
Breslow index	**0.017**	2.03 (0.75–5.50)	0.166	** < 0.0001**	1.80 (1.05–3.07)	**0.033**
Ulceration	** < 0.0001**	4.17 (1.42–12.22)	**0.009**	**0.008**	1.94 (1.12–3.36)	**0.019**
N stage	** < 0.0001**		**0.010**	** < 0.0001**		**0.002**
0		Reference			Reference	
1		2.88 (0.60–13.92)	0.188		1.50 (0.75–2.98)	0.249
2		5.04 (1.38–18.37)	**0.014**		2.05 (1.07–3.91)	**0.030**
3		10.42 (2.49–43.63)	**0.001**		4.02 (1.92–8.38)	** < 0.0001**
Resection margin	** < 0.0001**	0.26 (0.09–0.77)	**0.015**	0.196	1.07 (0.52–2.20)	0.865
Adjuvant therapy^#^	0.452	Not included		0.258	Not included	

^#^Adjuvant therapy include chemotherapy and high dose interferon. Bold font mean *p* value < 0.05. *OS* overall survival

**Table 4:** Univariate and multivariate DFS analysis of the patients in the acral and non-acral cutaneous melanoma

Variable	Disease-free survival					
	Non-acral cutaneous melanoma			Acral melanoma		
	Univariate analysis	Multivariate analysis		Univariate analysis	Multivariate analysis	
	*p*	HR (95% CI)	*p*	*p*	HR (95% CI)	*p*
Age	0.085	1.29 (0.63–2.67)	0.491	0.489	Not included	
Gender	0.144	0.49 (0.22–1.11)	0.087	0.282	Not included	
Breslow index	**0.028**	1.99 (0.97–4.10)	0.060	** < 0.0001**	1.91 (1.29–2.82)	**0.001**
Ulceration	** < 0.0001**	6.28 (2.58–15.28)	** < 0.0001**	0.545	Not included	
N stage	** < 0.0001**		** < 0.0001**	** < 0.0001**		** < 0.0001**
0		Reference			Reference	
1		7.33 (2.54–21.13)	** < 0.0001**		2.40 (1.45–3.98)	**0.001**
2		4.53 (1.63–12.58)	** < 0.0001**		2.74 (1.50–4.08)	** < 0.0001**
3		12.37 (4.43–34.61)	** < 0.0001**		3.82 (2.14–6.84)	** < 0.0001**
Resection margin	**0.002**	0.51 (0.21–1.25)	0.143	0.080	1.09 (0.64–1.86)	0.758
Adjuvant therapy^#^	**0.010**	0.88 (0.31–2.50)	0.809	0.474	Not included	

^#^Adjuvant therapy include chemotherapy and high dose interferon. Bold font mean *p* value < 0.05. *DFS* disease-free survival

### Acral melanoma

**Fig. 2:**
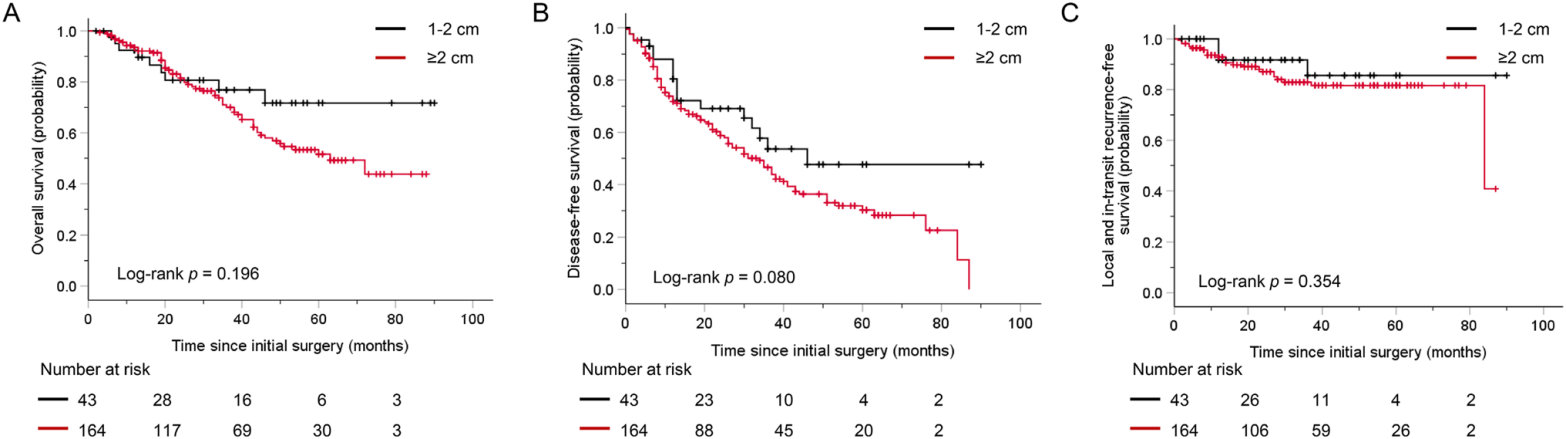
Kaplan–Meier plot curves in patients with acral melanoma with different resection margins. (**A**) Overall survival (*p* = 0.196). (**B**) Disease-free survival (*p* = 0.080). (**C**) Local and in-transit recurrence-free survival (*p* = 0.354)

Most acral melanoma patients were male (*n* = 120, 58%) and older than 60 years (*n* = 126, 60.9%). Ninety-four (45.4%) acral melanoma patients had thicker melanomas (Breslow thickness > 4 mm), and 114 (55.1%) had ulceration (Table 1). As shown in Table 2, 43 (20.8%) patients in the acral melanoma group had a 1–2-cm resection margin. Similar to the independent prognostic factors for the non-acral cutaneous melanoma prognosis, Breslow thickness, Clark level, ulceration and N stage contributed independently to the acral melanoma prognosis. Interestingly, resection margin was not correlated with OS (Table 3; *p* = 0.196 by log-rank analysis, *p* = 0.865 by multivariate survival analysis), DFS (Table 4; *p* = 0.080 by log-rank analysis, *p* = 0.758 by multivariate survival analysis) or LITRFS (Table 5; *p* = 0.354 by log-rank analysis) in acral melanoma (Fig. 2).

**Table 5:** Univariate and multivariate LITRFS analysis of the patients in the acral and non-acral cutaneous melanoma

Variable	Local and in-transit recurrence-free survival					
	Non-acral cutaneous melanoma			Acral melanoma		
	Univariate analysis	Multivariate analysis		Univariate analysis	Multivariate analysis	
	*p*	HR (95% CI)	*p*	*p*	HR (95% CI)	*p*
Age	0.066	1.81 (0.47–7.04)	0.392	0.996	Not included	
Gender	0.871	Not included		0.060	1.48 (0.96–2.29)	0.080
Breslow index	0.073	2.56 (0.63–10.43)	0.189	0.348	Not included	
Ulceration	**0.017**	4.66 (0.96–22.73)	0.057	0.545	Not included	
N stage	0.080		0.286	0.134		0.196
0		Reference			Reference	
1		3.60 (0.67–19.25)	0.134		2.23 (0.92–5.39)	0.077
2		1.57 (0.24–10.14)	0.635		1.86 (0.69–5.04)	0.223
3		4.82 (0.72–32.47)	0.106		0.50 (0.06–3.84)	0.503
Resection margin	**0.002**	0.19 (0.05–0.71)	**0.013**	0.354	Not included	
Adjuvant therapy^#^	0.320	Not included		0.312	Not included	

^#^Adjuvant therapy include chemotherapy and high dose interferon. Bold font mean *p* value < 0.05. *LITRFS* local and in-transit recurrence-free survival

## Discussion

This multicenter retrospective study aimed to investigate whether narrow-margin excision is warranted for thick acral melanoma, and the results were surprising. In the non-acral cutaneous melanoma group, patients with NCCN-recommended resection margins (> 2 cm) showed better local tumor control and longer survival than those who with narrow margins. However, for patients with acral melanoma, narrow resection margins of 1–2 cm did not reduce OS, DFS or LITRFS compared with recommended resection margins.

The resection margin of cutaneous melanoma has been discussed for more than a century. The earliest report recommended a 5-cm margin, which was described by Handley in 1907. The necessity of such extensive surgery has been questioned and has led to numerous prospective randomized clinical trials (RCTs) seeking to determine the optimal margin. The resection margin was first narrowed to 2 cm [17, 18] and then to 1 cm for thin melanomas (< 2 mm) [19]. Then, 2-cm margins were demonstrated to be safe for intermediate-thickness melanomas (1–4 mm) [20], and most recent trials for thicker melanomas (> 2 mm) have shown 2-cm margins to be safe (Swedish trial) [7, 21]. However, whether the resection margin can be reduced to 1 cm for thick melanoma remains controversial. MelMarT is a registered phase III surgical RCT comparing 1-cm versus 2-cm surgical margins for patients with primary cutaneous melanoma with a Breslow thickness > 1 mm [22]. While more patients in the 2-cm margin group required reconstruction and suffered from an increased wound necrosis rate, no differences in QoL were noted between groups. Another long-term follow-up RCT suggested that a 1-cm excision margin is inadequate for cutaneous melanoma thicker than 2 mm (UK trial) [23]. Additionally, ongoing RCTs, such as Melanoma Margins Trial II (MelMarT-II; ClinicalTrials.gov number, NCT03860883), are comparing the safety of 1-cm margins versus 2-cm margins for thick melanomas (stage > T2b). Unfortunately, major limitations exist in these RCTs. Except for MelMarT and MelMarT-II, the above trials accrued patients before SLNB became a standard surgical practice, and the absente of SLNB may influence the primary endpoints. Another limitation is that very few acral melanoma patients were enrolled in these RCTs. The Swedish trial and UK trial deliberately excluded acral subtypes. For comparison, all the patients enrolled in the current study had thick melanomas greater than 2 mm and received SLNB and/or CLND/TLND. To increase the robustness of the results, while a large sample of acral melanoma patients from multiple centers was investigated, we also enrolled the contemporaneous non-acral type as a control.

Several retrospective studies have discussed the resection margin in acral melanoma. One study in South Korea including 129 acral melanoma patients found that a 1-cm excision margin was safe for recurrence or mortality control in thin acral melanoma; for thick melanoma (> 1 mm, n = 76), a 2-cm resection margin provided improved local control but did not improve DFS or melanoma-specific survival (MSS) [24]. Except the limited number of participants in this study, notably, a Breslow thickness of 1 mm may not be an ideal cutoff point when deciding whether to retain a 2-cm resection margin. Another study in Japan retrospectively reviewed 100 acral melanoma patients, and among sixty-two T1–T3 melanoma patients, the mortality rates in the narrow-margin group and recommended-margin group were similar [15]. However, patients with T4 melanoma treated with narrow-margin excision had a higher mortality rate. Of note, among all the T4 melanomas, seven patients had a 5-mm resection margin, which may have contributed to the poor survival in the narrow-margin group. One promising method to safely reduce the resection margin may be Mohs micrographic surgery (MMS), which has shown considerable advantages for local recurrence and mortality control in both non-acral [25, 26] and acral melanomas [27]. Nevertheless, atypical melanoma cells are more difficult to identify in frozen sections than in paraffin sections, thus necessitating pathologists with extensive expertise. In addition, Mohs surgery substantially lengthens the operation time. All these factors cause certain resistance and difficulty in its promotion. Therefore, determining a definite range of resection margins may still be the easiest approach to follow.

In addition to the inherent drawbacks of all retrospective studies, one noted limitation is that no patients enrolled in this study had received targeted therapy or immunotherapy, due to the relatively late entry of relevant drugs into China.

## Conclusions

In conclusion, our study suggested that a resection margin ≥ 2 cm is necessary for non-acral cutaneous melanoma. However, a narrow resection margin of 1–2 cm may not decrease the OS, DFS or LITRFS of thick acral melanoma patients. Despite some limitations, the current study may provide a useful basis for further well-designed, multicenter RCTs.

## Data Availability

The datasets used or analysed during the current study are available from the corresponding author on reasonable request.
